# Burden of Ileal Perforations Among Surgical Patients Admitted in Tertiary Care Hospitals of Three Asian countries: Surveillance of Enteric Fever in Asia Project (SEAP), September 2016–September 2019

**DOI:** 10.1093/cid/ciaa1309

**Published:** 2020-12-01

**Authors:** Saqib H Qazi, Mohammad T Yousafzai, Nasir S Saddal, Irum F Dehraj, Rozina S Thobani, Afshan Akhtar, Jamal R Syed, Abdul M Kazi, Aneeta Hotwani, Najeeb Rahman, Junaid Mehmood, Jason R Andrews, Stephen P Luby, Denise O Garrett, Farah N Qamar

**Affiliations:** 1 Section of Pediatric Surgery, Department of Surgery, Aga Khan University, Karachi, Pakistan; 2 Department of Pediatrics and Child Health, Aga Khan University, Karachi, Pakistan; 3 National Institute of Child Health, Karachi, Pakistan; 4 Aga Khan University Medical College, Karachi, Pakistan; 5 Division of Infectious Diseases and Geographic Medicine, Stanford University, Stanford, California, USA; 6 Applied Epidemiology, Sabin Vaccine Institute, Washington, DC, USA

**Keywords:** enteric perforation, ileal perforation, *Salmonella* Typhi, enteric fever, typhoid fever

## Abstract

**Background:**

Typhoid fever is caused by *Salmonella enterica* subspecies enterica serovar Typhi (*S*. Typhi) and can lead to systemic illness and complications. We aimed to characterize typhoid-related ileal perforation in the context of the population-based Surveillance of Enteric Fever in Asia Project (SEAP) in Bangladesh, Nepal and Pakistan.

**Methods:**

Between September 2016 and September 2019, all cases of nontraumatic ileal perforation with a clinical diagnosis of typhoid were enrolled from 4 tertiary care hospitals in Karachi, 2 pediatric hospitals in Bangladesh, and 2 hospitals in Nepal. Sociodemographic data were collected from patients or their caregivers, and clinical and outcome data were retrieved from medical records. Tissue samples were collected for histopathology and blood cultures where available.

**Results:**

Of the 249 enrolled cases, 2 from Bangladesh, 5 from Nepal and 242 from Pakistan. In Pakistan, most of the cases were in the 0–15 (117/242; 48%) and 16–30 (89/242; 37%) age groups. In all countries, males were most affected: Pakistan 74.9% (180/242), Nepal 80% (4/5), and Bangladesh 100% (2/2). Blood culture was done on 76 cases; 8 (11%) were positive for *S*. Typhi, and all were extensively drug resistant (XDR) *S*. Typhi. Tissue cultures was done on 86 patients; 3 (3%) were positive for *S*. Typhi, and all were XDR *S*. Typhi, out of 86 samples tested for histopathology 4 (5%) revealed ileal perforation with necrosis. Culture or histopathology confirmed total 15 (11%) enteric fever cases with ileal perforation are similar to the clinically diagnosed cases. There were 16/242 (7%) deaths from Pakistan. Cases of ileal perforation who survived were more likely to have sought care before visiting the sentinel hospital (*P* = .009), visited any hospital for treatment (*P* = .013) compared to those who survived.

**Conclusions:**

Although surveillance differed substantially by country, one reason for the higher number of ileal perforation cases in Pakistan could be the circulation of XDR strain of *S*. Typhi in Karachi.

Typhoid fever caused by *Salmonella enterica* subspecies enterica serovar Typhi (*S*. Typhi) can lead to systemic illness that has been a major source of morbidity and mortality in low-income communities [1]. Despite advancements in medical treatment, addressing typhoid fever remains difficult in many parts of the world such as South Asia, where poor water supply and sanitation remain a major problem [2, 3]. In such settings, incidence can be as high ≥10 cases per 100 000 population per year, and 1 in 5 children experiences typhoid infection by the age of 10 years [4, 5]. An important factor in decreasing the burden of typhoid fever has been the emergence of antibiotic resistance [6]. Multidrug resistant (MDR) strains of *S*. Typhi, resistant to ampicillin, trimethoprim-sulfamethoxazole (TMP-SMZ), and chloramphenicol have increased dramatically in the last 2 decades [7]. More recently, extensively drug resistant (XDR) strains have been isolated in Pakistan that are resistant to ampicillin, TMP-SMZ, fluoroquinolones, and third-generation cephalosporins, leaving limited treatment options for typhoid treatment [8]. Untreated typhoid disease can lead to altered mental states (termed the typhoid state), ileus, gastrointestinal bleeding, ileal perforation septic shock, and death [1, 9, 10]. Ileal perforation is a fatal complication that usually occurs in the second or third week of illness, due to inflammation and necrosis of Peyer’s patches. However, it has also been reported in the second week of illness in lower middle-income countries [11–14]. Typhoid related ileal perforation has been reported in 0.8%–39% of typhoid patients [15–17] with a higher incidence in low- and middle-income countries [18, 19]. Reported mortality rates due to ileal perforation range from 5% in wealthy regions to 80% in poor regions [20, 21]. The burden of typhoid related ileal perforation has been attributed to these factors: increasing antibiotic resistance, delay in diagnosis, delay in surgical treatments, inappropriate surgical technique and nonavailability of guidelines for appropriate postoperative care [22]. The emergence of antimicrobial resistance, especially XDR strains of *S*. Typhi in Pakistan, may increase the risk of typhoid perforation. The lack of availability of antimicrobial sensitivity testing and limited understanding of its importance in most secondary level hospitals and first level care facilities can increase the delay in initiating the appropriate course of antimicrobial therapy and increase the risk of treatment failures.

With worsening antimicrobial resistance, it is important to assess the burden and consequences of typhoid related ileal perforation. We aim to describe the frequency of typhoid related ileal perforation and attributed mortality from a multicenter, prospective surveillance project in 3 Asian countries.

## METHODS

### Study Design and Settings

This prospective surveillance study was part of the Surveillance for Enteric Fever in Asia Project (SEAP) conducted at different hospitals and lab networks in Bangladesh, Nepal, and Pakistan from September 2016 to September 2019 [23]. For the enrollment of surgical cases in Bangladesh, the study was conducted in Dhaka Shishu (Children’s) Hospital and Shishu Shasthya Foundation Hospital. Both serve the pediatric population. In Nepal the study was conducted in Dhulikhel Hospital and Kathmandu Medical College and Teaching Hospital, which serves both adult and pediatric population. In Pakistan we selected JinnahPostgraduate Medical Center (JPMC), the National Institute of Child Health (NICH), Aga Khan University Hospital (AKUH),and Kharadar General Hospital (KGH). These included bothpublic and private tertiary care facilities serving adults, pediatric populations, or both. All site hospitals public and private were purposefully selected to include patients with diverse socioeconomic background and logistical accessible. Enrollment of typhoid-related ileal perforation cases was initiated in September 2016 at AKUH and KGH, in November 2016 at NICH, and in February 2018 at JPMC. In Nepal and Bangladesh, the enrollment was started in September 2016 at all of their sites.

### Eligibility Criteria

All hospitalized cases with a diagnosis of nontraumatic ileal perforation due to suspected typhoid were identified from the daily census sheets of the respective surgical units in the selected hospitals.

The diagnosis of typhoid perforation was made by the admitting surgeon and was mainly clinical, supplemented by intraoperative findings of acute, inflamed, terminal ileal perforation. Patients with ileal perforation due to tuberculosis and malignancy identified perioperatively or on subsequent tissue histopathology were excluded.

### Enrollment and Data Collection

Trained research associates with nursing or medical background approached the patient under the supervision of a surgeon used a structured questionnaire to collect sociodemographic data through a face-to-face interview with either the adult patient or the caregiver of a child. The clinical diagnosis, surgical record, and outcome were obtained from the patients’ medical records. The medical records were reviewed daily to identify any change in the diagnosis. Close coordination and liaison were established with all surgeons in the respective units of the selected hospitals. Detailed data on the sociodemographic and clinical characteristics was collected from all surgical enrolled patients from all countries. Information from the questionnaire and medical records was directly entered into the tablets. Follow-up of all patients was done through phone calls 6 weeks after enrollment. Information about outcome (survival/death) and recovery was collected during 6 week follow-up.

### Data Management

The tablets’ electronic data collection system automatically assigned a unique identification number to the data of each participant. The research supervisor cross-checked each questionnaire for any errors or an inconsistent response at the end of the day before syncing the data into the central database.

### Laboratory Testing and Procedures

After enrollment of the patient, blood culture was collected and then treatment was initiated. The trained research staff collected 5–10 mL of the blood sample and submitted to clinical laboratory of AKUH for testing. During surgery, the operating surgeon obtained tissue samples in normal saline and provided them to the study staff for histopathology testing. The study staff delivered the tissue samples to the Infectious Diseases and Research Laboratory (IDRL) of Aga Khan University for culture and histopathology testing. Ultrasound or any other tests needed for the confirmation of the perforation were performed at the discretion of the operating surgeon.

### Data Analysis

Data were retrieved directly from the server, and descriptive statistics were calculated for both continuous and categorical variables. For continuous variables, the mean with standard deviation were calculated. For categorical variables, frequency with percentages was calculated. Factor analysis of ileal perforation patients’ household possessions was performed to determine socioeconomic status (SES). Factors with high eigenvalues were considered to classify SES and generate scores. These were then divided into 3 quintiles. Trends of ileal perforation across different age groups were analyzed. A comparison of the recovered versus those who died was analyzed. The number of ileal perforation across different age groups were plotted in a line graph, and comparison of the survivors versus nonsurvived was analyzed using Pearson χ ^2^ or Fisher exact test to calculate the *P* values. A *P* value of < .05 was considered statistically significant. In order to evaluate whether culture or histopathology confirmed enteric fever cases with ileal perforation are similar to the clinically diagnosed cases, we performed a sensitivity analysis as reported in Supplementary Table 1. *P* values were calculated using χ ^2^ or Fisher exact test for categorical and independent sample *t* test for quantitative variables.

### Ethical Consideration

Trained research associates with nursing or medical background approached the patients and/or their attendants and informed consent and/or assent was sought before the enrollment. We obtained written informed consent from all adult patients (age 18 years and above). For all patients aged < 11 years consent was taken from their parents or legal guardians. For patients aged 11–17 years, assent along with parental consent was also obtained. Each individual participant had the right to withdraw her/his consent at any time. Confidentiality was maintained by the assigning unique identifier number, and no personal identifying details such as name was obtained. The study was approved by the ethical review committees (ERC) of the Bangladesh Institute of Child Health, Nepal Health Research Council, Aga Khan University, National Bioethics committee (NBC) Pakistan and Stanford University.

## RESULTS

During September 2016 to September 2019 a total of 249 patients with ileal perforation were enrolled: 242 from Pakistan, 2 cases from Bangladesh, and 5 from Nepal. In all 3 countries males were most affected: Pakistan 180/242 (74.9 %); Bangladesh 2/2 (100%); and Nepal 4/5 (80%). In Pakistan, 54% (130/242) of typhoid related ileal perforation were enrolled during 2018 and 30% in 2019 (73/242). Of the typhoid-related ileal perforation cases 41% (99/242) were in the first SES quintile, and 39% (94/242) were in the third quintile (Table 1).

**Table 1.** Sociodemographic and Clinical Characteristics of Patients With Enteric Perforation in Pakistan, Nepal, and Bangladesh, Surveillance for Enteric Fever in Asia Project (SEAP), 2016–2019 (N = 249)

Sociodemographic Variables	Pakistan	Nepal	Bangladesh
	N = 242 (%)	N = 5 (%)	N = 2 (%)
Age in years			
0–15	117 (48)	1 (20)	2 (100)
16–30	89 (37)	1 (20)	0 (0)
>30	36 (15)	3 (60)	0 (0)
Sex			
Male	180 (75)	4 (80)	2 (100)
			
Clinical characteristics	Median (IQR)		
Duration of illness before hospitalization	11 (6,18)		
Duration of hospitalization	8 (6,13)		
Fever	234 (97)		
Abdominal pain	224 (93)		
Constipation/diarrhea	137 (57)		
Vomiting	148 (61)		
Health-seeking behavior			
Patient sought any care from somewhere else before enrollment visit	219 (91)		
Treatment from any hospital	163 (67)		
Treatment from pharmacy, clinic or physician	134 (55)		
Received treatment from traditional healer	10 (4)		
Prior treatment	**N** = **208**		
Antibiotic	141 (58)		
Antipyretic	194 (80)		
Analgesic	123 (51)		
Antidiarrheal	16 (7)		
Patients receiving antibiotics during hospitalization	**N** = **236 (%)**		
Type of antibiotics			
Cephalosporin	86 (36)		
Piperacillin and tazobactam	23 (10)		
Ciprofloxacin	34 (14)		
Metronidazole	33 (14)		
Carbapenem	34 (14)		
Other	26 (11)		
Chest X-ray	**N** = **242 (%)**		
Performed	177 (73)		
Indicated but not performed/ missing/not indicated	65 (27)		
Abdominal ultrasound finding	**N** = **146 (%)**		
Internal bleeding/intestinal hemorrhage	17 (12)		
Intestinal perforation	114 (78)		
Hepatomegaly/splenomegaly	23 (16)		
Peritonitis	70 (48)		
Other	54 (37)		
Blood culture positive for *S.* Typhi	8/76 (11)	1/3 (33)	
MDR	0	0	
XDR	8 (100)	0	
Tissue culture	**N** = **86 (%)**		
NICH	57 (66)		
JPMC	29 (34)		
Tissue culture positive for *S*. Typhi	3 (3)		
Histopathology confirmed Ileal perforation with necrosis	4 (5)		
Blood/ tissue culture/ histopathology positive for *S*. Typhi (individuals)	15/131 (11)		
Hospital where surgery performed	**N** = **242 (%)**		
AKUH/KGH	19 (8)		
NICH	103 (42)		
JPMC	120 (50)		
Final outcome at discharge	**N** = **242 (%)**	**n** = **5 (%)**	**n** = **2 (%)**
Recovered	226 (93)	5 (100)	2 (100)
Died	16 (7)	0 (0)	0 (0)
Social and economic status			
Wealth status scores (tertile)	N = 242 (%)		
Low wealth status	99 (41)		
Medium wealth status	49 (20)		
High wealth status	94 (39)		
Year of surveillance	**N** = **242 (%)**		
2016	4 (2)		
2017	35 (14)		
2018	130 (54)		
2019	73 (30)		

Abbreviations: AKUH, Aga Khan University Hospital; JPMC, Jinnah Postgraduate Medical Center; IQR, interquartile range; KGH, Kharadar General Hospital; MDR, multidrug resistant; NICH, National Institute of Child Health; XDR, extensively drug resistant.

The median (interquartile range [IQR]) duration of illness prior to hospitalization was 11 (6, 18) days, and the duration of hospitalization was 8 (6, 13) days. Fever, abdominal pain, constipation/diarrhea, and vomiting were the common clinical characteristics of the patients: 91% (219/242) sought care prior to hospitalization and 58% (142/242) self-reported that they had received an antibiotic before presenting to the hospital. Blood culture was done on 76 cases; 8 (11%) were positive for *S*. Typhi and all were XDR *S*. Typhi. Tissue culture was done on 86 patients; 3 (3%) were positive for *S*. Typhi, and all were XDR *S*. Typhi. Culture or histopathology confirmed 15 (11%) ileal perforation cases were statistically similar to clinically diagnosed ileal perforations in terms of sociodemographic characteristics, clinical symptoms at the time of presentation, and final outcome at discharge. Only the occurrence of 1 or more clinical symptoms was statistically significant (Supplementary Table 1). Of the typhoid related ileal perforation cases from Pakistan, there were 16/242 (7%) deaths; 10 were aged 0–15 years. Among recovered patients, the median and IQR of the duration of illness prior to hospitalization was 11 (6, 17); of those who died it was 15 (10, 28) days.

**Figure 1.**
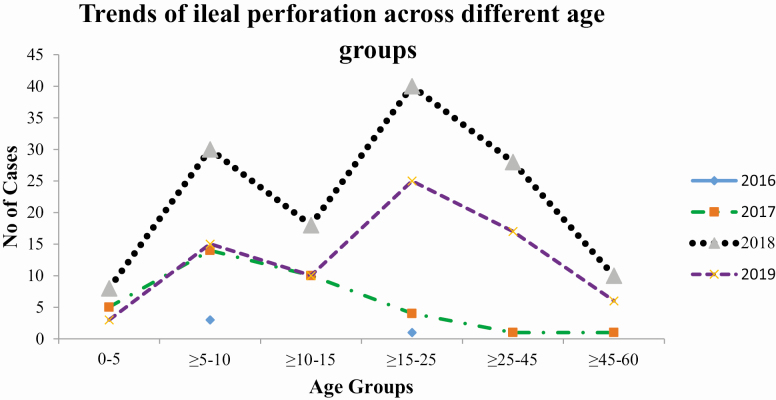
Trend of ileal perforation across different age groups, Karachi, Pakistan, Surveillance for Enteric Fever in Asia Project (SEAP), 2016–2019.

Cases of typhoid related ileal perforation who recovered as compared to those who died were more likely to seek care before visiting the sentinel hospital (92% vs 69%; *P* = .009) and more likely to visit other hospitals for treatment (68% vs 63%; *P* = .013). No other significant difference in terms of clinical or sociodemographic characteristics between the two groups were identified (Table 2). The frequency of ileal perforation was highest in all age groups in 2018, followed by 2019 and 2017. The pattern of typhoid related ileal perforation was similar in 2018 and 2019, with highest frequency in the age group 15–25 followed by 5–10 years old; however, in 2017 the highest number of ileal perforation occurred among 5–10 years old age group only (Figure 1).

**Table 2.** Comparison of Patients With Enteric Perforation Who Died Versus Those Who Survived in Pakistan, Surveillance for Enteric Fever in Asia Project (SEAP), 2016–2019

Sociodemographic Variables	Recovered	Died	*P* value
	N = 226 (%)	N = 16 (%)	
Age in years			
0–15	107 (48)	10 (62)	.035
16–30	87 (38)	2 (13)	
>30	32 (14)	4 (25)	
Sex			
Male	171 (76)	9 (56)	.086
Female	55 (24)	7 (44)	
			
Clinical characteristics	Median (IQR)	Median (IQR)	
Duration of illness before hospitalization	11 (6,17)	15 (10,28)	.635
Duration of hospitalization	8 (6,13)	8 (4,19)	.488
	N (%)		
Fever	219 (97)	15 (94)	.495
Abdominal pain	208 (92)	16 (100)	.502
Constipation / diarrhea	123 (54)	14 (88)	.010
Vomiting	134 (59)	14 (88)	.081
Health-seeking behavior			
Patient sought any care from somewhere else before enrollment visit	208 (92)	11 (69)	.009
Received treatment from any hospital	153 (68)	10 (63)	.013
Received treatment from any pharmacy, clinic, and physician	125 (55)	9 (56)	.942
Received treatment from traditional healer	9 (4)	1 (6)	.019
Prior treatment			
Patient received antibiotic	132 (58)	9 (56)	.338
Patient received antipyretic and analgesic	185 (82)	12 (75)	.496
Patient received antidiarrheal prior	15 (7)	1 (6)	.354
Patients receiving antibiotics during hospitalization	221 (98)	15 (94)	.164
Type of antibiotics			
Cephalosporin	31 (14)	3 (20)	.590
Piperacillin and tazobactam	79 (36)	7 (47)	.575
Ciprofloxacin	33 (15)	1 (7)	.430
Metronidazole	33 (15)	0 (0)	.136
Carbapenem	24 (11)	2 (13)	.793
Other	21 (10)	2 (13)	.665
Chest X-ray	N = 226 (%)	N = 16 (%)	
Performed	163 (72)	14 (88)	.180
Indicated, but not performed/ missing and not indicated	63 (28)	2 (12)	
Abdominal ultrasound performed	136 (60.2)	10 (62.5)	.854
Ultrasound finding			
Internal bleeding/intestinal hemorrhage	16 (12)	1 (10)	.867
Intestinal perforation	107 (79)	7 (70)	.522
Hepatomegaly and splenomegaly	20 (15)	3 (30)	.200
Peritonitis	67 (49)	3 (30)	.239
Blood culture			
*S.* Typhi	8 (11)	0	.426
Hospital where surgery performed	N = 226 (%)	N = 16 (%)	
AKUH/KGH	19 (8)	0 (0)	.247
NICH	94 (42)	9 (56.3)	.486
JPMC	113 (50)	7 (43.8)	.775
Social and economic status			
Wealth status scores (tertile)	N = 226 (%)	N = 16 (%)	
Low wealth status	92 (41)	7 (44)	.723
Medium wealth status	47 (21)	2 (13)	
High wealth status	87 (38)	7 (44)	

Abbreviations: AKUH, Aga Khan University Hospital; IQR, interquartile range; JPMC, Jinnah Postgraduate Medical Center; KGH, Kharadar General Hospital; NICH, National Institute of Child Health.

## DISCUSSION

We conducted surveillance in three large cities in Bangladesh, Nepal, and Pakistan for ileal perforation. The surveillance in Karachi included 2 additional hospitals specifically to look for perforations. Compared with Bangladesh and Nepal, we observed more ileal perforation cases in Pakistan, at a time when there was widespread circulation of XDR typhoid. Due to the unavailability of sensitive tool for the diagnosis of typhoid-related ileal perforation, in this study all cases were diagnosed based on intraoperative findings supplemented with clinical sign and symptoms. Blood cultures were sent by the treating physicians in only a small proportion of the cases; the tissue samples either were not collected at the time of surgery, or tissue samples were collected in formalin hindering culture. Only a minority cases of ileal perforation were confirmed through blood and tissue cultures and histopathology testing. Traditionally, diagnosis is made mainly on the basis of clinical history and examination, X-ray abdomen, and ultrasound abdomen. Although serologic and bacteriologic data may be supportive, these are frequently negative. Blood cultures are positive for *S.* Typhi in only 3–34% of cases of typhoid perforation, and cultures of the stool and peritoneal fluid are usually negative for this organism [24]. Although surgical diagnosis of typhoid related ileal perforation is not 100% sensitive or specific, this is the most frequently reported method for the diagnosis of typhoid-related ileal perforation in the existing literature. Most of the earlier studies reported from African countries were based on confirmation on Widal test or clinical suspicion, which is known to have poor sensitivity and specificity [25–28]. Polymerase chain reaction (PCR) based testing and immunohistochemical staining of the tissue as a tool for diagnosis of typhoid-related ileal perforation is currently under investigation and might improve the diagnosis in future.

Differences in the surveillance sites could partly explain the high number of cases from Pakistan. Although the SEAP surveillance sites were used to enroll ileal perforation cases from Bangladesh and Nepal, in Pakistan (in addition to the SEAP surveillance sites) 2 additional public sector tertiary care referral hospitals were used. Being public sector referral hospitals, patients were from lower socioeconomic backgrounds and also from the very remote areas of Sindh province. Another contributing explanation to the larger number of cases in Pakistan could be the difference in antibiotic resistance patterns, especially XDR strains of *S*. Typhi in the 3 countries. Pakistan is the only country among the SEAP sites where XDR typhoid fever is prevalent Yousafzai et al Pakistan budren paper in this supplement (S214). In addition, the minority of the blood samples from the patients with ileal perforation from Pakistan from which *S.* Typhi were isolated, all were XDR. Therefore, a higher proportion of XDR typhoid cases in Pakistan might have contributed to the higher number of typhoid-related ileal perforation. Whether the higher number of ileal perforation cases in Pakistan as compared to the other 2 countries is the function of XDR outbreak of *Salmonella* Typhi in Pakistan or just the reflection of how and where the surveillance was conducted in these 3 countries is hard to conclude [29]. A 2-year surveillance study in Tanzania during 2006–2008 enrolled 104 typhoid-related ileal perforation cases. The study reported male preponderance and higher incidence in the age group 11–20 years old [26]. In contrast, a study from India during 2015–2017, reported higher proportions of typhoid-related ileal perforation among adults aged 31–40 years [30]. Nonetheless, males being at higher risk of typhoid-related ileal perforation is consistent with reports from India and several other countries [31, 32]. Only 1 study from Nigeria reported a higher number of ileal perforation cases among female populations [33]. The exact mechanism behind male preponderance for ileal perforation is not known. However, there might be a genetic predisposition among males that heightens the risk of ileal perforation [34]. Another reason could be the higher proportion of culture confirmed typhoid cases among the male population as compared to females. SEAP data from Pakistan showed significantly higher proportion of culture confirmed typhoid among the male population Yousafzai et al paper in this supplement.

In this study, the duration of illness among patients with ileal perforation was >10 days before hospitalization. Typhoid-related ileal perforation classically presents in the second week of illness, and hospitalization for typhoid-associated ileal perforation is normally long, ranging from 2 to 4 weeks. The duration of hospitalization is dependent on the condition of the patient at the time of admission and postoperative complications [33, 35–37].

Studies from other countries also reported delayed presentation and poor socioeconomic status as an important predictor of enteric perforations and its associated mortality [22]. In this study, 16 deaths (7%) were observed among typhoid-related ileal perforation in Pakistan. Duration of illness, socioeconomic status, and prior use of antibiotics was not associated with mortality. However, mortality was significantly higher among children (<15 years old). The reported mortality ranged from 4% in northern part of India to as high as 34% in parts of sub-Saharan Africa [33, 38]. Mortality associated with enteric perforations can be prevented with early diagnosis and treatment, access to surgical care, availability of better surgical facilities, trained surgeons, and prevention of postoperative complications [39]. Unfortunately, limitations in healthcare in general and surgical care in particular is lacking in lower and middle-income countries and can result in delays in accessing care and subsequent peri- or postoperative mortalities [22].

Our study had several limitations; a larger number of cases were identified from Pakistan presumably because this was the only site that had 2 surgical units of large public sector hospitals included as sentinel sites for enrollment. Only a subset of the cases could be confirmed through blood cultures and histopathological examination of the tissues, and hence some misclassification might have occurred. However, clinical suspicion of typhoid based on a history of fever for more than a week, followed by perforation was used as a criterion for clinical suspicion of typhoid-related ileal perforation and the intraoperative findings of the surgeons verified the diagnosis. The absence of a sensitive tool to confirm typhoid-related ileal perforation might have resulted in some classification bias resulting in over-estimation of cases in this study. However, the study team worked closely with the operating surgeons to minimize any bias in diagnosis. Patients with ileal perforation due to tuberculosis and malignancy identified perioperatively or on subsequent tissue histopathology were excluded. Patients with typhoid-related ileal perforation who presented as dead or who died immediately after reaching the emergency department of the respective hospitals and could not be operated or diagnosed were not accounted for in this study, and hence the case fatality rate might have been underestimated.

## CONCLUSIONS

Cases of typhoid-related ileal perforation were identified mainly in Pakistan; however, because of lack of availability of sensitive diagnostic tools, a bacteriologic diagnosis was not possible in most cases. Early diagnosis and treatment are essential for adequate management of ileal perforation in typhoid endemic countries. The development of improved tools to specify the cause of intestinal perforation would permit better understanding and tracking of this important occasional consequence of *Salmonella* typhoid and paratyphoid infection.

## Supplementary Data

Supplementary materials are available at *Clinical Infectious Diseases* online. Consisting of data provided by the authors to benefit the reader, the posted materials are not copyedited and are the sole responsibility of the authors, so questions or comments should be addressed to the corresponding author.

ciaa1309_suppl_Supplementary_Table_1

## Notes

***Financial support.*** This work was supported by Bill & Melinda Gates Foundation (Investment ID 008335). S. P. L. and J. A. report grants from Gates Foundation, during the conduct of the study.

***Supplement sponsorship.*** This supplement is sponsored by the Sabin Vaccine Institute and made possible by a grant from the Bill & Melinda Gates Foundation.

***Potential conflicts of interest.*** The authors: No reported conflicts of interest. All authors have submitted the ICMJE Form for Disclosure of Potential Conflicts of Interest.
