# Supplementary material for: Burden of Ileal Perforations Among Surgical Patients Admitted in Tertiary Care Hospitals of Three Asian countries: Surveillance of Enteric Fever in Asia Project (SEAP), September 2016–September 2019
Source: Clin Infect Dis. 2020 Dec 1;71(Suppl 3):S232–8. doi: 10.1093/cid/ciaa1309 (PMC7705870; doi:10.1093/cid/ciaa1309)
Supplement: ciaa1309_suppl_Supplementary_Table_1 [file ciaa1309_suppl_supplementary_table_1.docx]

**Supplementary Table 1.**

**Comparison of culture or histopathology confirmed ileal perforations with clinically diagnosed ileal perforations.**

|  | **IP Culture or histopath confirmed** | | **IP with clinical diagnosis** | | **Total** | | **p-value** |
| --- | --- | --- | --- | --- | --- | --- | --- |
|  | **N=15** | **%** | **N=227** | **%** | **N=242** | **%** |  |
| **Age in years** | | | | | | | |
| 0-15 | 8 | 53.33 | 109 | 48.02 | 117 | 48.35 | 0.65 |
| 16-30 | 6 | 40.00 | 83 | 36.56 | 89 | 36.78 |  |
| >30 | 1 | 6.67 | 35 | 15.42 | 36 | 14.88 |  |
| **Gender** | | | | | | | |
| male | 13 | 86.67 | 167 | 73.57 | 180 | 74.38 | 0.26 |
| female | 2 | 13.33 | 60 | 26.43 | 62 | 25.62 |  |
| **Fever** | | | | | | | |
| yes | 14 | 93.33 | 220 | 96.92 | 234 | 96.69 | 0.45 |
| no | 1 | 6.67 | 7 | 3.08 | 8 | 3.31 |  |
| **Abdominal Pain** | | | | | | | |
| yes | 12 | 80.00 | 212 | 93.39 | 224 | 92.56 | <0.001 |
| no | 1 | 6.67 | 15 | 6.61 | 16 | 6.61 |  |
| doesn't know | 2 | 13.33 | 0 | 0.00 | 2 | 0.83 |  |
| **Constipation/Diarrhea** | | | | | | | |
| Yes | 7 | 46.67 | 130 | 57.27 | 137 | 56.61 | 0.42 |
| No | 8 | 53.33 | 97 | 42.73 | 105 | 43.39 |  |
| **Vomiting** | | | | | | | |
| yes | 5 | 33.33 | 143 | 63.00 | 148 | 61.16 | 0.005 |
| no | 9 | 60.00 | 83 | 36.56 | 92 | 38.02 |  |
| doesn't know | 1 | 6.67 | 1 | 0.44 | 2 | 0.83 |  |
| **Wealth Index quintiles** | | | | | | | |
| Low SES | 5 | 33.33 | 94 | 41.41 | 99 | 40.91 | 0.79 |
| Middle SES | 3 | 20.00 | 46 | 20.26 | 49 | 20.25 |  |
| High SES | 7 | 46.67 | 87 | 38.33 | 94 | 38.84 |  |
| **Final outcome at discharge** | | | | | | | |
| Recovered | 15 | 100.00 | 211 | 92.95 | 226 | 93.39 | 0.29 |
| died | 0 | 0.00 | 16 | 7.05 | 16 | 6.61 |  |
|  | | | | | | | |
| XDR | 8 | 100 | 0 | 0 | 8 | 100 |  |
|  | **Median (IQR)** | | **Median (IQR)** | | **Median (IQR)** | |  |
| Duration of illness before hospitalization | 12 (4-17) | | 10 (6-17) | | 11 (6-18) | | 0.90 |
| Duration of hospitalization | 8 (6-12) | | 8 (6-14) | | 8 (6-13) | | 0.96 |

*P values calculated based on chi-square or fisher exact tests.*
